# Species of the genus *Lamachus* Förster (Hymenoptera, Ichneumonidae) parasitizing diprionid sawflies (Hymenoptera, Diprionidae) with descriptions of two new species and a key to Chinese species

**DOI:** 10.3897/zookeys.249.4069

**Published:** 2012-12-10

**Authors:** Tao Li, Mao-Ling Sheng, Shu-Ping Sun

**Affiliations:** 1 The Key Laboratory for Silviculture and Conservation of Ministry of Education, Beijing Forestry University, Beijing 100083, P. R. China; 2 General Station of Forest Pest Management, State Forestry Administration, 58 Huanghe North Street, Shenyang 110034, P. R. China

**Keywords:** Ctenopelmatinae, *Lamachus*, new species, parasitoid, Diprionidae, *Neodiprion huizeensis*, *Diprion jingyuanensis*, biology

## Abstract

Four species of the genus *Lamachus* Förster 1869 belonging to the tribe Mesoleiini of the subfamily Ctenopelmatinae (Hymenoptera, Ichneumonidae) are reported from China. Two of them are new to science, *Lamachus nigrus* Li, Sheng & Sun, **sp. n.** and *Lamachus rufiabdominalis* Li, Sheng & Sun, **sp. n.** were reared from *Neodiprion huizeensis* Xiao & Zhou, in Guizhou Province of the Oriental part of China. The biology of *Lamachus rufiabdominalis* is described. A key to the species of *Lamachus* known from China is provided.

## Introduction

*Lamachus* Förster 1869, belonging to the subfamily Ctenopelmatinae of Ichneumonidae (Hymenoptera), comprises 24 described species (Yu et al. 2012), of which four are known from the Eastern Palearctic, 14 from the Western Palearctic and six from the Nearctic. Hitherto, most hosts of this genus are sawflies of the families Diprionidae and Tenthredinidae, including *Diprion* Schrank, *Neodiprion* Rohwer, *Gilpinia* Benson and *Pristiphora* Latreille (Cushman 1937, 1939; Finlayson and Finlayson 1958; Meyer 1936, Morris et al. 1937; Price and Tripp 1972; Townes 1970; Uchida 1955; Yu et al. 1997).

Two species of *Lamachus* Förster have been known in China (Sheng and Sun 2007; Li et al. 2012): *Lamachus gilpiniae* Uchida, parasitizing *Diprion jingyuanensis* Xiao & Zhang (Hymenoptera, Diprionidae) (Li et al. 2012), found in Shanxi Province, China, which is a parasitoid of *Gilpinia tohi* Takeuchi (Hymenoptera, Diprionidae) in Japan (Uchida 1955); and *Lamachus sheni* Sheng & Sun 2007, from Henan Province, China.

In the last four years the first author has been researching web-spinning and leaf-rolling sawflies in China, and has reared large numbers of ichneumonids. New discoveries will be reported successively. In this article, two new species of *Lamachus* are reported.

## Materials and methods

Materials used were collected using the following methods.

**Rearing parasitoids.** Cocoons of sawflies were collected under the naturally heavily infested trees in Weining County (26°54'N, 104°13'E, elevation 2000 to 2200 m), Guizhou Province, and stored individually in glass tubes (100 mm long and 15 mm in diameter) with a piece of filter paper dipped in distilled water (to prevent desiccation), plugged with absorbent cotton, and maintained in the laboratory at room temperature. The emerged insects were collected daily.

**Direct collection.** Specimens were collected using entomological sweep nets in the forests, where the trees were naturally heavily infested by sawflies.

Images of whole insects were taken using a CANON Power Shot A650 IS. Other images were taken using a Cool SNAP 3CCD attached to a Zeiss Discovery V8 Stereomicroscope and captured with QCapture Pro version 5.1. Morphological terminology is based on Gauld (1991). Wing vein nomenclature is based on Ross (1936) and the terminology of Mason (1986, 1990).

Type specimens are deposited in the Insect Museum, General Station of Forest Pest Management (GSFPM), State Forestry Administration, P. R. China.

### Descriptions

#### Genus Lamachus

Förster, 1869

http://species-id.net/wiki/Lamachus

Lamachus Förster, 1869. Verhandlungen des Naturhistorischen Vereins der Preussischen Rheinlande und Westfalens, 25(1868): 206. Type species: *Tryphon lophyrum* Hartig.

##### Diagnosis.

Clypeus small, almost flat or weakly convex. Upper tooth of mandible slightly longer than or equal to lower tooth. Notaulus absent, or short and weak. Propodeal carinae weak or absent. Areolet present. Fore wing with vein 1cu-a distal of 1/M by about 0.25 times length of 1cu-a. Hind wing vein 1-cu longer than cu-a. Glymma present. Median dorsal carina of first tergum weak; dorsolateral carina complete. Setae of female genital plate slanted backward.

##### Key to species of *Lamachus* known in China

**Table:** 

1	Median longitudinal carina of propodeum distinct. Median dorsal carina of first tergum distinct. Upper tooth of mandible as long as lower tooth	*Lamachus gilpiniae* Uchida
–	Median longitudinal carina of propodeum absent (Figs 4, 11). Median dorsal carina of first tergum incomplete or absent. Upper tooth of mandible slightly longer than lower tooth	2
2	Third to fifth terga red (Fig. 7). First tergum 2.5 times as long as apical width. Second tergum 0.8 times as long as apical width (Fig. 12). Antenna with 46–48 flagellomeres	*Lamachus rufiabdominalis* Li, Sheng & Sun, sp. n.
–	All terga black. First tergum 1.4 or 2.1 times as long as apical width. Second tergum 0.6 or 1.1 times as long as apical width. Antenna with 38(39) to 40 flagellomeres	3
3	First tergum 2.1 times as long as apical width, median dorsal carina absent. Second tergum 1.1 times as long as apical width. Scutellum and postscutellum black. Basal 0.7 of hind tibia white	*Lamachus sheni* Sheng & Sun
–	First tergum 1.4 times as long as apical width, basal portion of median dorsal carina present. Second tergum 0.6 times as long as apical width (Fig. 6). Lateral and apical portions of scutellum and entire postscutellum yellow. Hind tibia black	*Lamachus nigrus* Li, Sheng & Sun, sp. n.

#### Lamachus nigrus

Li, Sheng & Sun sp. n.

urn:lsid:zoobank.org:act:02E9E94E-0D92-43FB-8A87-64F6138A2687

http://species-id.net/wiki/Lamachus_nigrus

Figures 1–6

##### Etymology.

The specific name is derived from the body being entirely black.

##### Types.

*Holotype*, female, CHINA: Weining, Guizhou Province, 13 March 2012, leg. Tao Li, Mao-Ling Sheng. *Paratypes*: 7 females and 5 males, CHINA: Weining, Guizhou Province, 8 to 24 March 2012, leg. Tao Li, Mao-Ling Sheng.

##### Diagnosis.

Malar space 0.4 to 0.5 times as long as basal width of mandible. Postocellar line approximately 1.5 to 1.6 times as long as ocular-ocellar line. Antenna with 39 to 40 flagellomeres. Fore wing with vein 1cu-a distal of 1/M by about 0.5 times length of 1cu-a. Vein 2-Cu approximately 1.5 times as long as 2cu-a. Hind wing vein 1-cu about 1.5 times as long as cu-a. First tergum 1.4 times as long as apical width. Abdomen entirely black.

##### Description.

**Figures 1–6.**
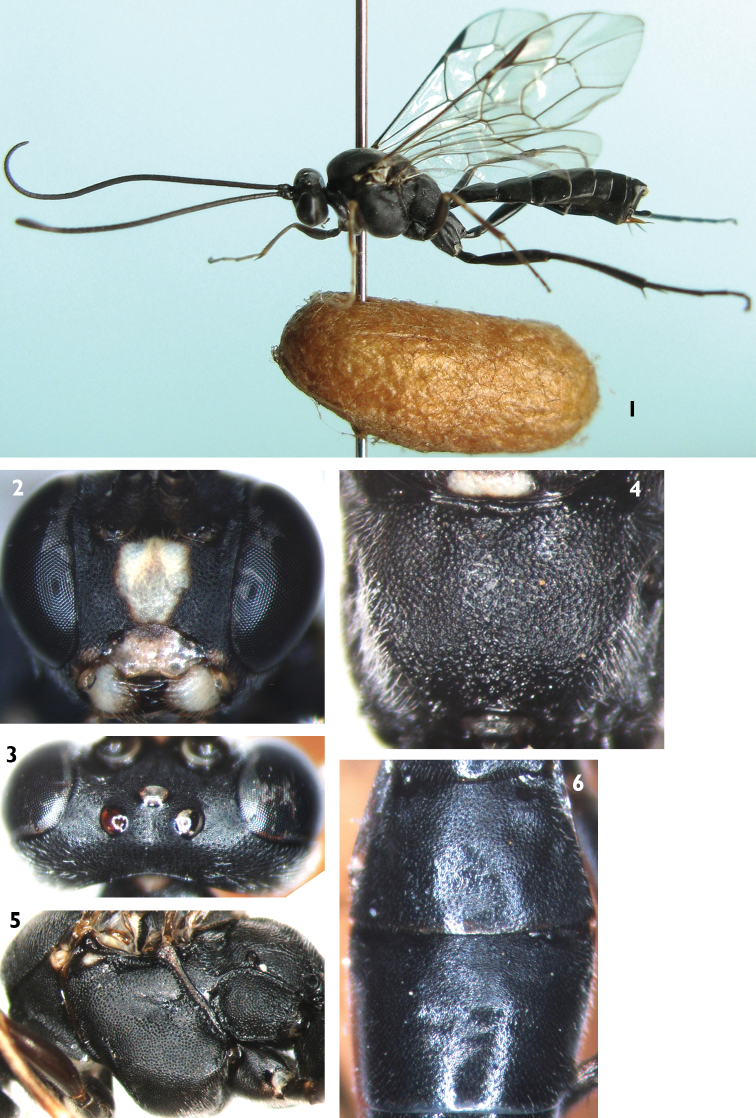
*Lamachus nigrus* Li, Sheng & Sun, sp. n. Holotype. Female **1** Body, lateral view **2** Head, anterior view **3** Head, dorsal view **4** Propodeum **5** Mesopleuron **6** Terga 2 to 3, dorsal view.

Female (Fig. 1). Body length 8.0 to 10.0 mm. Fore wing length 7.5 to 9.0 mm.

**Head.** Inner eye orbits weakly concave at level of antennal insertions. Face (Fig. 2) 1.6 to 1.7 times as wide as long, with dense punctures, upper center margin with weak longitudinal wrinkles. Clypeus approximately flat and smooth, with sparse setae, 2.0 times as wide as long; central part of apical margin distinctly concave. Mandible smooth, with weak punctures, upper tooth slightly longer than lower tooth. Malar space with fine leathery texture, 0.4 to 0.5 times as long as basal width of mandible. Gena with evenly dense punctures. Posterior part of vertex (Fig. 3) with texture as that of gena. Ocellar triangle with fine leathery texture, with weak median longitudinal groove. Postocellar line about 1.5 to 1.6 times as long as ocular-ocellar line. Frons approximately flat, with texture as that of face, lower portion concave at antennal areas. Antenna with 39 to 40 flagellomeres, ratio of length from first to fifth flagellomeres: 9.0:5.0:4.5:4.5:4.0. Occipital carina complete.

**Mesosoma.** Anterior portion of pronotum with fine leathery texture and dense punctures; median portion with short transverse wrinkles; upper posterior portion with dense punctures. Mesoscutum evenly convex, with texture as that of upper posterior portion of pronotum. Notaulus evident on anterior half of mesoscutum. Scutoscutellar groove wide, with weak longitudinal wrinkles. Scutellum evenly convex, with sparse punctures, larger than those of mesoscutum. Postscutellum transverse, punctures denser than on scutellum. Mesopleuron (Fig. 5) evenly convex, with punctures as scutellum. Epicnemial carina weak, 0.5 times as long as mesopleuron. Speculum with fine leathery texture. Metapleuron convex, with dense punctures and fine wrinkles. Submetapleural carinae complete. All tibiae with distinct pegs. Ratio of length of hind tarsomeres 1:2:3:4:5 is 25.0:11.0:7.0:4.0:6.0. Fore wing with vein 1cu-a distal of 1/M by about 0.5 times length of 1cu-a. Vein 2-Cu approximately 1.5 times as long as 2cu-a. Fore wing with stalked triangular areolet. Vein 3rs-m distinctly longer than 2rs-m. Areolet receiving vein 2m-cu approximately at lower-posterior angle. Hind wing vein 1-cu about 1.5 times as long as cu-a. Propodeum (Fig. 4) evenly convex, without areas, with texture as that of mesoscutum. Pleural carina distinct. Propodeal spiracle approximately circular, located at anterior 0.3 of propodeum.

**Metasoma.** First tergum 1.4 times as long as apical width, with fine granulose texture. Basal portion of median dorsal carinae present. Spiracle circular, at middle of first tergum. Second tergum (Fig. 6) approximately 0.6 times as long as apical width, with texture as that of first tergum. Thyridium present. Third tergum (Fig. 6) and following terga slightly compressed, with fine leathery texture and dense small punctures. Ovipositor sheath approximately 0.3 times apical depth of metasoma. Ovipositor with dorsal notch. Basal portion of ovipositor very wide, apically portion distinctly slender.

**Color** (Fig. 1)**.** Black, except the following. Median portion of face, clypeus, mandible except black teeth, ventral portion of fore coxa, median portion of subalar ridge, hind corner of pronotum, lateral and apical portions of scutellum, postscutellum, yellow. Maxillary and labial palpi blackish brown. Anterior portion of fore femur, tibia, tarsomeres, basal half of mid tibia, yellowish brown. Apical portion of mid tibia and tarsus blackish brown. Wing membrane brownish hyaline. Pterostigma and veins brownish black.

**Male.** Body length about 7.0 to 9.0 mm. Fore wing length about 6.0 to 7.0 mm. Antenna with 37 to 39 flagellomeres. Median and lower lateral portions of face, clypeus, mandible (teeth black), maxillary palp, anterior portion of fore coxa, hind corner of pronotum, subalar ridge, basal portion of notaulus, lateral fleck of scutellum, postscutellum, yellow. Labial palp, pterostigma, veins, blackish brown. Fore (except median portion of tibia blackish brown, first to fourth tarsomeres yellowish brown) and mid legs dark brown.

##### Host.

*Neodiprion huizeensis* Xiao & Zhou (Hymenoptera: Diprionidae).

##### Host plant.

*Pinus armandi* Franch. (Pinaceae).

##### Biology.

The mature larva forms a cocoon inside host’s cocoon and outside the body of the host larva.

##### Remarks.

This new species is similar to *Lamachus gilpiniae* but can be distinguished from the latter by the following combination of characters: upper tooth of mandible longer than lower tooth; postocellar line approximately 1.5 to 1.6 times as long as ocular-ocellar line; median longitudinal carina of propodeum absent; first tergum 1.4 times as long as apical width; second tergum 0.6 times as long as apical width. *Lamachus gilpiniae*: upper tooth of mandible as long as lower tooth; postocellar line approximately as long as ocular-ocellar line; median longitudinal carina of propodeum distinct; first tergum 1.7 times as long as apical width; second tergum 0.7 to 0.8 times as long as apical width.

#### Lamachus rufiabdominalis

Li, Sheng & Sun sp. n.

urn:lsid:zoobank.org:act:8FE1BA4D-4DD7-436F-B9FA-A06AC082D196

http://species-id.net/wiki/Lamachus_rufiabdominalis

Figures 7 15

##### Etymology.

The specific name is derived from the red metasoma.

##### Types.

*Holotype*, female, CHINA: Weining, Guizhou Province, 14 March 2012, leg. Tao Li, Mao-Ling Sheng. *Paratypes*: 51 females and 26 males, CHINA: Weining, Guizhou Province, 3 March to 15 April 2012, leg. Tao Li, Mao-Ling Sheng.

##### Diagnosis.

Malar space 0.5 times as long as basal width of mandible. Postocellar line as long as ocular-ocellar line. Antenna with 46 to 48 flagellomeres. Fore wing with vein 1cu-a slightly distal of 1/M. Vein 2-Cu approximately as long as 2cu-a. Hind wing vein 1-cu about as long as cu-a. First tergum 2.5 times as long as apical width. Median and apical portions of second tergum, third to fifth terga red.

##### Description.

**Figures 7–12.**
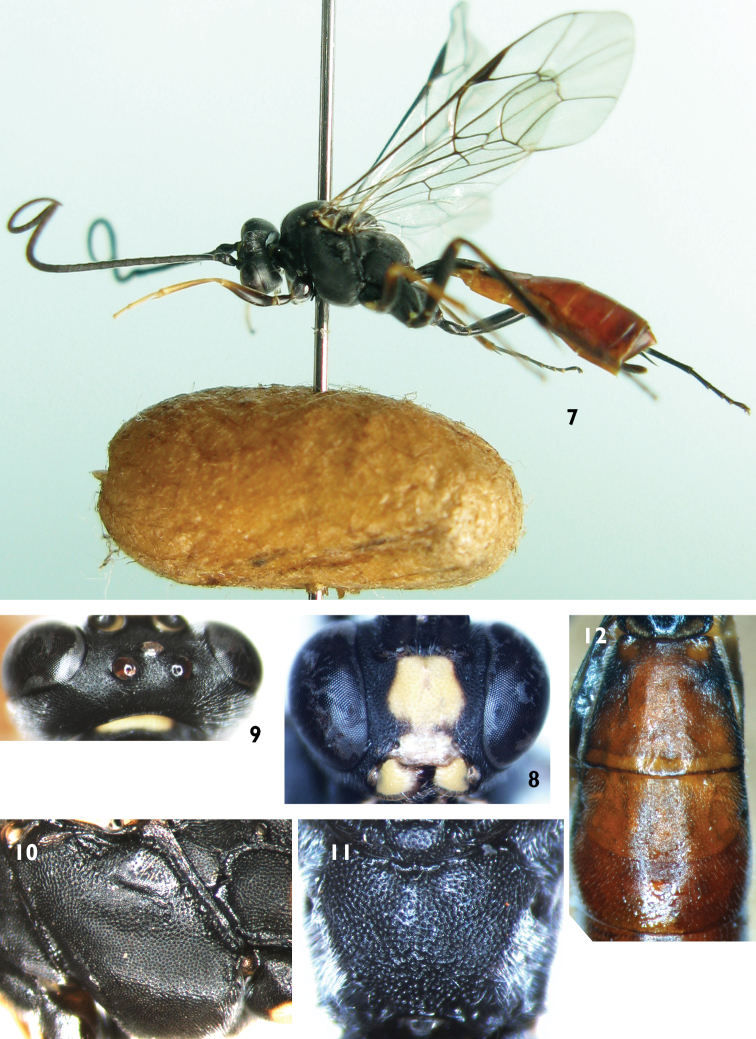
*Lamachus rufiabdominalis* Li, Sheng & Sun, sp. n. Holotype. Female **7** Body, lateral view **8** Head, anterior view **9** Head, dorsal view **10** Mesopleuron **11** Propodeum **12** Terga 2 to 3, dorsal view.

Female (Fig. 7). Body length 7.0 to 10.0 mm. Fore wing length 7.0 to 9.0 mm.

**Head.** Inner eye orbits weakly concave at level of antennal insertions. Face (Fig. 8) 0.9 times as wide as long, with dense punctures, upper center with median longitudinal groove. Clypeus smooth, weakly convex at basal portion, 2.5 times as wide as long; apical portion distinctly concave, with fine wrinkles. Mandible smooth, with fine punctures, upper tooth slightly longer than lower tooth. Malar space with fine leathery texture and dense punctures, 0.5 times as long as basal width of mandible. Gena with texture as that of malar space. Vertex (Fig. 9) smooth, with fine leathery texture. Ocellar triangle weakly convex. Postocellar line about equal to ocular-ocellar line. Middle portion of frons evenly convex, with texture as that of vertex. Lateral portion of frons evenly concave. Antenna with 46 to 48 flagellomeres, ratio of length from first to fifth flagellomeres: 10.0:6.0:6.0:6.0:5.0. Occipital carina complete.

**Mesosoma.** Anterior portion of pronotum with fine leathery texture and dense punctures; upper part of median portion with weak wrinkles; lower part of median portion with dense punctures; upper posterior portion with dense punctures. Mesoscutum evenly convex, with dense punctures. Notaulus weak. Scutoscutellar groove wide, with weak longitudinal wrinkles. Scutellum evenly convex, with texture as that of mesoscutum. Postscutellum transverse, punctures finer than on scutellum. Middle and lower portions of mesopleuron (Fig. 10) convex, with texture as that of mesoscutum. Upper portion of mesopleuron with rough punctures. Speculum small, with fine granulose texture. Lower portion of speculum weakly concave. Metapleuron evenly convex, with texture as that of mesopleuron. Submetapleural carina complete. Ratio of length of hind tarsomeres 1:2:3:4:5 is 10.0:5.0:3.5:2.0:2.0. Fore wing with vein 1cu-a weakly outside of 1/M. Vein 2-Cu approximately as long as 2cu-a. Fore wing with stalked triangular areolet. Vein 3rs-m distinct longer than 2rs-m. Areolet receiving vein 2m-cu approximately at lower-posterior angle. Hind wing vein 1-cu about as long as cu-a. Propodeum (Fig. 11) evenly convex, without areas, with texture as that of mesoscutum. Propodeal spiracle circular, located at about anterior 0.3 of propodeum.

**Metasoma.** First tergum 2.5 times as long as apical width, with fine leathery texture and sparsely punctate. Spiracle circular, small, located at middle of first tergum. Dorsolateral carina complete posterior to spiracle. Ventrolateral carina complete. Second tergum (Fig. 12) approximately 0.8 times as long as apical width, with texture as that of first tergum and apical portion sparsely punctate. Thyridium circular. Ovipositor sheath approximately 0.3 times as long as hind tibia. Ovipositor with dorsal notch. Basal portion of ovipositor very wide. Apical portion distinctly slender.

**Color** (Fig. 7)**.** Black, except the following. Middle portion of face (width of fleck 0.75 times as long as that of face in holotype, width of fleck 0.60 to 0.86 times as long as width of face among individuals), clypeus, mandible except black teeth, front portion of fore coxa and first trochanter, part of anterior of mid coxa, hind corner of pronotum, fleck of propodeum, yellowish green. Anterior side of fore femur, tibia and tarsus, apical portion of mid femur, tibia and tarsus, yellowish brown. Hind tibia entirely black, or subbasally with a small, indistinctly yellowish spot. Central and apical portion of second tergum, third to fifth terga, red. Pterostigma and veins brownish black. Wings brownish hyaline.

**Male.** Body length about 7.0 to 9.0 mm. Fore wing length about 5.0 to 7.0 mm. Antenna with 48 flagellomeres. Face, coxa and front portion of trochanters of fore leg, coxa and front portion of trochanters of mid leg, yellowish green. Hind tibia entirely black. Other characteristics as for female.

##### Host.

*Neodiprion huizeensis* Xiao & Zhou (Hymenoptera: Diprionidae).

##### Host plant.

*Pinus armandi* Franch. (Pinaceae).

##### Remarks.

This new species is similar to *Lamachus iwatai* Momoi 1962, but can be distinguished from the latter by the following combination of characters: first tergum 2.5 times as long as apical width; hind tarsomere 4 as long as tarsomere 5; inner orbit, malar space and mesoscutum entirely black. *Lamachus iwatai*: first tergum 1.7 times as long as apical width; hind tarsomere 4 shorter than tarsomere 5; inner orbit, malar space, a median spot of mesoscutum, yellow.

##### Biology.

**Figures 13–15.**
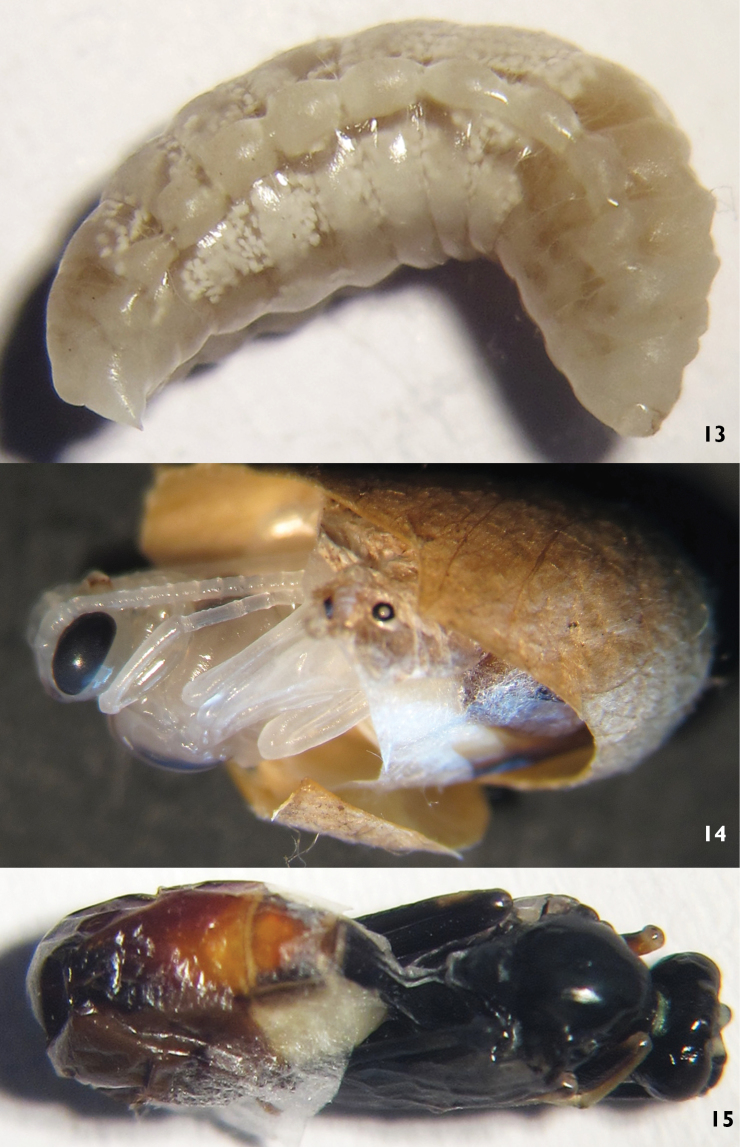
*Lamachus rufiabdominalis* Li, Sheng & Sun, sp. n. **13** Larva 1**4,15** Pupa.

*Lamachus rufiabdominalis* is an endoparasitoid of *Neodiprion huizeensis* larvae. The mature larva of *Lamachus rufiabdominalis* is cream-colored (Fig. 13), the color changing continuously as development continues. The body of the pupa is yellowish white, compound eyes and ocelli, red. After four days, the compound eyes became black, ocelli changed to reddish brown and the teeth red (Fig. 14). One day later, the ventral profile of the mesothorax, anterior portion of median lobe and lateral portion of lateral lobe of mesoscutum, were brown. After two days, the median portion of the face and mandible (teeth, blackish brown) were yellowish white, femur yellowish brown, most of the first tergum (except apically reddish brown) were blackish brown, second and third terga yellowish brown with reddish marks. The body was black, antenna blackish brown, median portion of face and basal portion of mandible yellowish green, femora yellowish brown, second and third terga red when the pupa was mature (Fig. 15). Of 78 adults of *Lamachus rufiabdominalis* that emerged from cocoons of *Neodiprion huizeensis*, the female to male ratio was 2.1:1. The parasitism rates of *Neodiprion huizeensis* by *Lamachus rufiabdominalis* were 1.2% to 1.3%. Adults of *Lamachus rufiabdominalis* emerged between 3rd and 30th March under laboratory conditions.

#### Lamachus gilpiniae

Uchida, 1955

http://species-id.net/wiki/Lamachus_gilpiniae

Figure 16

Lamachus gilpiniae Uchida, 1955. Insecta Matsumurana, 19: 3.

##### Specimens examined.

2 females, CHINA: Taiyuan, Shanxi Province, 22 September 2009. 2 females, CHINA: Taiyuan, Shanxi Province, 25 May to 1 June 2010, Mao-Ling Sheng; 1 male, CHINA: Taiyuan, Shanxi Province, 26 July 2010, Tao Li.

##### Host.

*Diprion jingyuanensis* Xiao & Zhang, *Gilpinia tohi* Takeuchi (Hymenoptera: Diprionidae) (Uchida 1955; Li et al. 2012).

##### Host plant.

*Pinus tabulaeformis* Carr. (Pinaceae).

**Figure 16.**
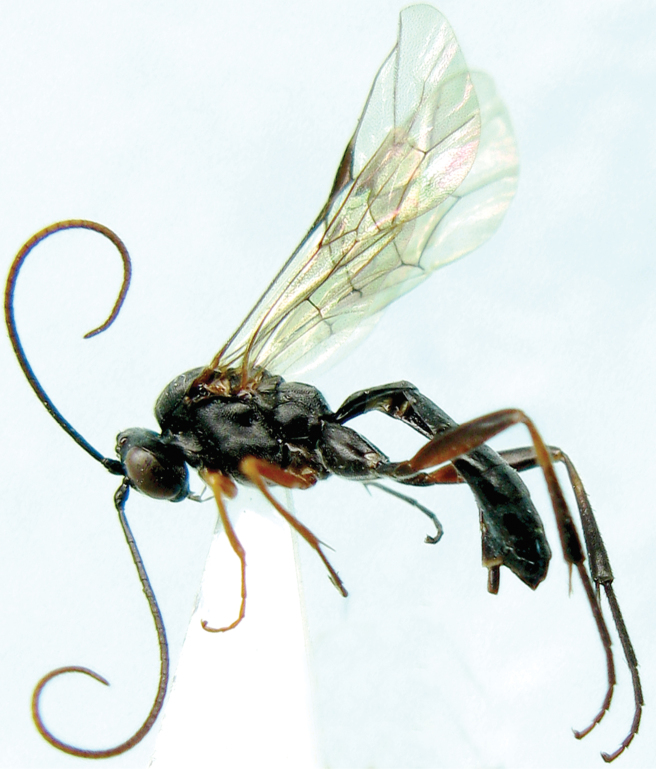
*Lamachus gilpiniae* Uchida, 1955. Female. Body, lateral view.

#### Lamachus sheni

Sheng & Sun, 2007

http://species-id.net/wiki/Lamachus_sheni

Figure 17

Lamachus sheni Sheng & Sun, 2007. Acta Zootaxonomica Sinica, 32(4):959.

##### Specimens examined. 

2 females, CHINA: Neixiang National Natural Reserve, Henan Province, 10 May 2006, Xiao-Cheng Shen.

##### Host.

Unknown.

**Figure 17.**
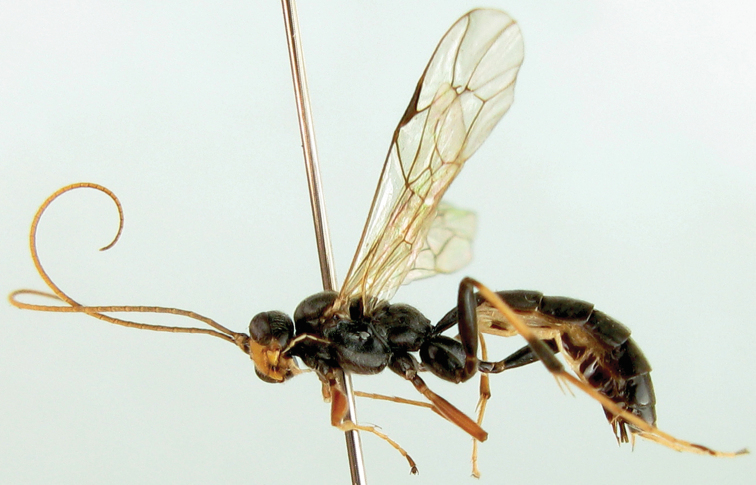
*Lamachus sheni* Sheng & Sun, 2007. Holotype. Female. Body, lateral view.

## Supplementary Material

XML Treatment for Lamachus

XML Treatment for Lamachus nigrus

XML Treatment for Lamachus rufiabdominalis

XML Treatment for Lamachus gilpiniae

XML Treatment for Lamachus sheni
